# The size-dependent influence of palladium doping on the structures of cationic gold clusters†

**DOI:** 10.1039/d1na00587a

**Published:** 2021-09-21

**Authors:** Piero Ferrari, Laia Delgado-Callico, Olga V. Lushchikova, Gao-Lei Hou, Francesca Baletto, Joost M. Bakker, Ewald Janssens

**Affiliations:** Quantum Solid-State Physics, Department of Physics and Astronomy, KU Leuven Leuven Belgium piero.ferrari@kuleuven.be; Department of Physics, King's College London London UK; Radboud University, Institute for Molecules and Materials, FELIX Laboratory Nijmegen The Netherlands

## Abstract

The physicochemical properties of small metal clusters strongly depend on their precise geometry. Determining such geometries, however, is challenging, particularly for clusters formed by multiple elements. In this work, we combine infrared multiple photon dissociation spectroscopy and density functional theory calculations to investigate the lowest-energy structures of Pd doped gold clusters, PdAu_*n*−1_^+^ (*n* ≤ 10). The high-quality experimental spectra allow for an unambiguous determination of the structures adopted by the clusters. Our results show that the Pd–Au interaction is so large that the structures of PdAu_*n*−1_^+^ and Au_*n*_^+^ are very different. Pd doping induces a 2D to 3D transition at much smaller cluster sizes than for pure Au_*n*_^+^ clusters. PdAu_*n*−1_^+^ clusters are three-dimensional from *n* = 4, whereas for Au_*n*_^+^ this transition only takes place at *n* = 7. Despite the strong Au–Pd interaction, the Au_*n*−1_^+^ cluster geometries remain recognizable in PdAu_*n*−1_^+^ up to *n* = 7. This is particularly clear for PdAu_6_^+^. In PdAu_8_^+^ and PdAu_9_^+^, Pd triggers major rearrangements of the Au clusters, which adopt pyramidal shapes. For PdAu_4_^+^ we find a geometry that was not considered in previous studies, and the geometry found for PdAu_8_^+^ does not correspond to the lowest-energy structure predicted by DFT, suggesting kinetic trapping during formation. This work demonstrates that even with the continuous improvement of computational methods, unambiguous assignment of cluster geometries still requires a synergistic approach, combining experiment and computational modelling.

## Abstract 2

The physicochemical properties of small metal clusters strongly depend on their precise geometry.

## Graphical abstract

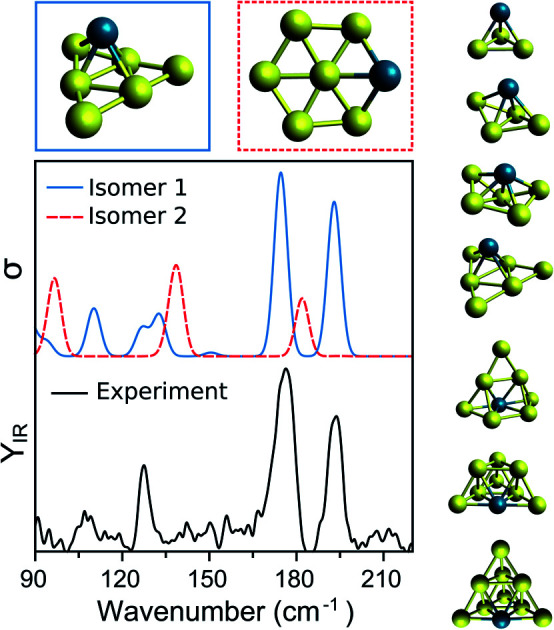

## Introduction

The physical and chemical properties of small metal clusters depend largely on the geometries adopted by these particles. For example, it has been predicted that different isomers of Au_*n*_^*q*^ (*n* ≤ 11, *q* = 0, ±1) clusters adsorb and dissociate H_2_ differently, with the lowest-energy structures being not necessarily more reactive towards hydrogen.^1^ Moreover, the shape of the cluster and its electronic level sequence are strongly entangled, as it is particularly clear for the known planar magic clusters^2^ and oblate species like Ag_15_^+^.^3^ Furthermore, the reactivity of Al_*n*_^−^ clusters has been shown to be size-dependent; Al_12_^−^ is very reactive towards methanol and water, while Al_13_^−^ is inert. Al_13_^−^ adopts an icosahedral geometry, with an isotropic distribution of the total −1e charge. Instead, Al_12_^−^ can either donate charge to or accept it from the molecules, increasing binding energies and reducing reaction barriers.^4^ Therefore, understanding the physical and chemical properties of a cluster requires precise knowledge about the adopted geometry.

Determining the geometry of a cluster, however, is challenging even if the system is composed of only a few atoms. For instance, there has been an intensive debate about the geometries adopted by small Au_*n*_^+^ clusters, with contradictory claims about the planar or three-dimensional structure of Au_8_^+^, that was only recently solved.^5^ Even for a cluster as small as Au_4_^+^, evidence of a higher energy isomer present in molecular beams was shown not long ago.^6^ These challenges are further exacerbated in bimetallic species, with an increasing number of possible configurations, making it difficult to locate with high certainty the global minimum on the potential energy surface, based purely on theory.^7^ Moreover, even if the global minimum is identified (for the specific theoretical method used), there is no guarantee that this is the isomeric structure in the experiment, as formation conditions may favour other isomers.^8^ Hence, assigning a cluster geometry requires the combination of dedicated experiments with precise quantum chemical calculations.^6,9–11^

The geometries of gold clusters have fascinated the scientific community for decades. Remarkable structures have been identified, like the tetrahedral (pyramidal) shape of neutral Au_20_,^12^ and the hollow cages of anionic gold clusters.^13^ Moreover, Au clusters are known for remaining planar up to relatively large sizes; the largest planar neutral gold cluster, for example, is Au_11_.^5,14,15^ For cationic Au_*n*_^+^, this transition size is *n* = 8, with three-dimensional and planar Au_8_^+^ isomers coexisting in molecular beams.^5^ These unique structures play a decisive role in defining properties such as relative stabilities,^16,17^ reactivities,^18,19^ de-excitation mechanisms,^20,21^ and optical responses.^22,23^

To the best of our knowledge, experimental determination of the geometries adopted by doped cationic gold clusters has so far only been achieved for YAu_*n*−1_^+^ (*n* ≤ 7)^24^ and the small AgAu_2,3_^+^ clusters.^25^ In the first case, Y doping induces a significant rearrangement of the gold cluster geometries, while in the second case, Ag acts as a substitutional dopant, where the symmetry of the cluster remains unchanged. Other doped Au_*n*_^+^ clusters have been studied by theoretical means, predicting a dopant-dependent structural influence. Density functional theory (DFT) calculations on MAu_*n*−1_^+^ (M = Ti, Fe) clusters indicate substitutional doping in the *n* ≤ 8 size range.^26^ Similarly, calculations of MAu_5_^+^ (M = Sc, Ti, Cr, and Fe)^27^ and AgAu_*n*−1_^+^ (*n* ≤ 18)^28^ predict substitutional doping. Instead, for YAu_*n*−1_^+^ (*n* ≤ 15) clusters there are significant rearrangements of Au_*n*_^+^ upon doping,^29^ just as for BeAu_*n*−1_^+^ (*n* ≤ 9).^30^

Pd doping significantly alters the properties of Au clusters. For example, it changes the size-to-size stability pattern of Au_*n*_^+^, where clusters with an odd number of atoms (even number of itinerant electrons) are experimentally found to be more stable.^17^ In PdAu_*n*−1_^+^, instead, *n* ≤ 8 clusters are experimentally found to be more stable for odd *n* values (even total number of atoms), while even *n* Pd doped cationic gold clusters are more stable for *n* > 8.^31^ This change in the stability pattern with size is attributed to Au always delocalizing its 6s electron in PdAu_*n*−1_^+^, whereas Pd can delocalize a 4d-electron depending on cluster size and cluster structure. Pd doping can also affect the cluster reactivity, illustrated by the increased CO and O_2_ binding energies on the doped clusters, compared to bare Au_*n*_^+^.^32,33^ For CO adsorption, the increase in binding energy becomes more pronounced from *n* = 7, as determined by a combination of mass spectrometry and statistical modelling.^32^ Furthermore, Pd doping was shown to quench optical absorption cross sections in the visible range.^34,35^ Finally, Pd is nowadays considered as a suitable dopant element in monolayer protected Au clusters, with diverse size-selected species synthesized in the laboratory.^36,37^

In this work, we unambiguously determine the geometry that PdAu_*n*−1_^+^ (*n* ≤ 10) clusters adopt in a molecular beam, by combining infrared multiple photon dissociation (IRMPD) spectroscopy experiments with extensive density functional theory calculations. Our results show that Pd induces a transition from planar to three-dimensional structures on the Au_*n*_^+^ clusters, with PdAu_*n*−1_^+^ adopting three-dimensional configurations from PdAu_3_^+^. The exact influence of Pd doping, however, is size-specific. Moreover, our work illustrates that dedicated experiments must be combined with accurate theoretical modelling for unambiguous assignment of cluster geometries.

## Methods

### Experiment

A molecular beam of Pd doped Au clusters is produced by laser ablation of bulk pure metal targets in a dual-target dual-laser source, using He gas for condensation.^38^ Complexes of cationic clusters with Ar are formed by adding 2% of Ar to the He carrier gas while the source is kept at 200 K. A 2 mm diameter skimmer initially shapes the cluster beam, followed by a 0.45 mm slit aperture. The cluster size distribution is irradiated by the laser light of the free-electron laser FELICE, adapted for intracavity experiments,^39^ and subsequently probed by a perpendicularly extracted reflectron time-of-flight mass spectrometer. The high pulse energy and large interaction volume reachable at FELICE allows for recording infrared spectra of high signal-to-noise ratio.

IR spectra are obtained by calculating the IRMPD yield, defined as the natural logarithmic ratio ln(*I*_0_/*I*(*ν*)) of cluster–Ar complex intensities in mass spectra without (*I*_0_) and with (*I*(*ν*)) the IR irradiation at laser frequency *ν*.^5^ The PdAu_*n*−1_^+^ clusters have high dissociation energies and are therefore difficult to fragment with the infrared light. Thus, Ar atoms are used as messenger species to record depletion spectra, since Ar has relatively low adsorption energies in PdAu_*n*−1_^+^Ar_*m*_ and the Ar loss channel can be used as a probe for resonant infrared absorption. This approach has been successfully followed in the past, allowing, in combination with computed infrared spectra of different isomers, the structural identification of diverse clusters.^40–42^ Unavoidably, the drawback of this method is that the recorded IR spectra are those of PdAu_*n*−1_^+^Ar_*m*_, whose metal framework could have a slightly different geometry from the bare PdAu_*n*−1_^+^ clusters. This possibility will be considered case by case. In the following, the infrared spectra of PdAu_*n*−1_^+^Ar_*m*_ clusters are discussed for *n* = 3–10, except for *n* = 6. For this size, infrared spectra could only be recorded for PdAu_5_^+^Ar_7_ (see Fig. S12 in the ESI†); such a large number of attached Ar atoms does not allow a proper analysis of the geometry adopted by the bare cluster.

### Calculations

DFT calculations are performed with the NWChem 6.8 software package,^43^ using the LC-ωPBEh functional in combination with the def2-TZVPP basis set. All electrons are included in the calculations for Ar and def2-ECP pseudopotentials are used for Au and Pd (19 and 18 valence electrons are included explicitly, respectively). Relativistic effects are accounted for by the pseudopotentials. Harmonic vibrational frequencies are calculated after a tight geometry optimization of each isomer, and the simulated infrared spectra are constructed by assuming Gaussian functions around each calculated frequency, with a full width at half maximum of 5 cm^−1^. To select the use of LC-ωPBEh, the IR spectrum of the PdAu_2_^+^Ar_6_ complex was used as benchmark, given the undoubtable triangular shape adopted by the metal PdAu_2_^+^ framework.

**Fig. 1:**
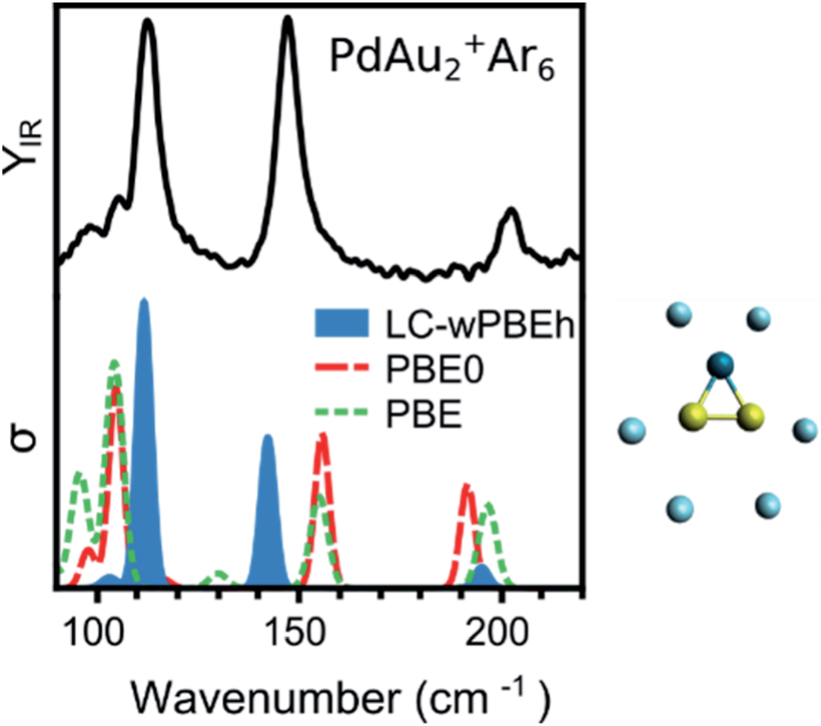
Experimental (top) and calculated (bottom) IR spectra of PdAu_2_^+^Ar_6_. Three functionals have been considered for the calculations (PBE, PBE0, and LC-ωPBEh), with LC-ωPBEh providing the best agreement. The cluster structure is depicted on the right of the figure. Gold, palladium, and argon atoms are represented by yellow, dark blue, and light blue spheres, respectively.

The IRMPD spectrum in Fig. 1 has three intense bands, at 113, 147, and 203 cm^−1^, corresponding to (mostly) the Au–Au stretch, the Au_2_–Pd stretch and the PdAu_2_ breathing modes, respectively. The measurement is compared to computed spectra using three functionals: PBE (GGA), PBE0 (hybrid), and LC-ωPBEh (long-range separated hybrid). A more extensive benchmarking using additional functionals is presented in the ESI (Fig. S1†). The best agreement is found by using the LC-ωPBEh functional, which does not need a scaling factor, as commonly applied in IRMPD studies,^12,24^ to accurately reproduce the positions of the three bands. There is only a slight red shift for the 147 cm^−1^ band. A quantification of the agreement is presented later. A small frequency scaling factor (1.04) is required to account for anharmonicity for increasing cluster sizes (*n* ≥ 4). This scaling factor was determined for pure Au_*n*_^+^·Ar_*m*_ clusters (using the same experimental conditions).^5^ Given the results of Fig. 1, the LC-ωPBEh functional is chosen for further calculations. This functional has also been used to calculate properties of Pd and Ag doped Au clusters,^25,31,32,44,45^ and correctly predicted the vibrational modes of Au^+^·Ar_*m*_ and Pd^+^·Ar_*m*_ complexes.^46^ An extensive isomer global search of PdAu_*n*−1_^+^ isomers was performed in ref. 31. Therefore, in the current work we consider the first six lowest-energy isomers identified in ref. 31 (except for PdAu_4_^+^; see the Results section for details) and we add the number of *m* Ar atoms as measured in the molecular beam. The Ar atoms are initially positioned on all possible atop coordination sites, followed by geometry optimization. Other coordination sites were considered but these were always found much higher in energy, as seen previously for Au_*n*_^+^.^47^

## Results

**Fig. 2:**
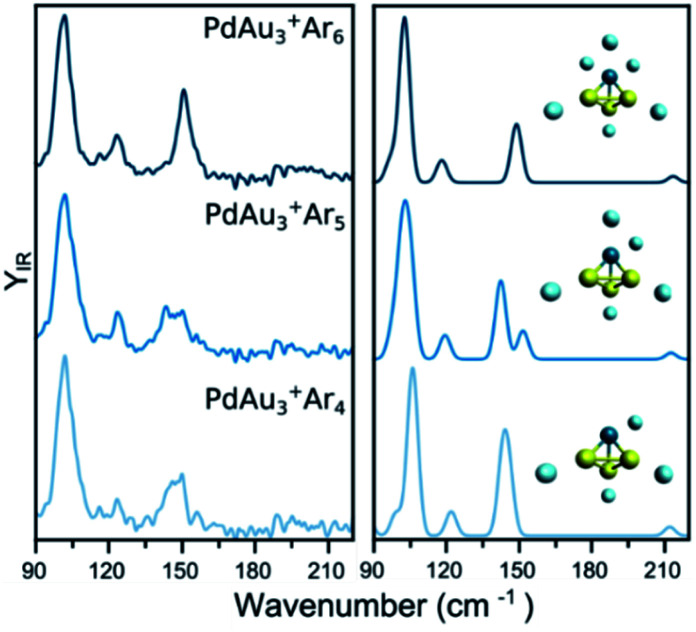
(left) Experimental IR spectra of PdAu_3_^+^Ar_*m*_ (*m* = 4, 5, 6) clusters. (right) Simulated IR spectra of the complexes, considering the lowest-energy isomer (see Fig. 5 for details).

The IR spectra of PdAu_3_^+^Ar_*m*_ (*m* = 4–6) are shown in the left panel of Fig. 2. The spectrum of PdAu_3_^+^Ar_4_ has two clear bands at 102 and 147 cm^−1^, in addition to a weaker one at 123 cm^−1^, and possible low-intensity bands around 180–210 cm^−1^. The interaction of Ar with PdAu_3_^+^ is relatively strong (calculated adsorption energy around 0.2 eV), even when four argon atoms are attached to the cluster (see Table S1 in the ESI†). Still, this energy is much smaller than the 2.17 eV corresponding to the fragmentation energy of the bare PdAu_3_^+^ cluster.^31^ Nevertheless, Ar participates in the vibrational modes of the complex and thus, it cannot be considered as a spectator atom that does not disturb the IR spectrum. This raises the pertinent question whether Ar affects the structure of the metal framework. To address this point, Fig. 2 also contains the measured spectra of PdAu_3_^+^Ar_5_ and PdAu_3_^+^Ar_6_, which show many similarities with that of PdAu_3_^+^Ar_4_. The spectral similarities strongly suggest that, for these complexes, extra Ar atoms do not significantly change the geometry of the metal cluster.

The experimental data of PdAu_3_^+^Ar_*m*_ (*m* = 4–6) is compared with simulated IR spectra using the putative lowest-energy structure for the metal framework (isomer 1), which adopts a 3D pyramidal geometry (other isomers are discussed later). The calculated IR spectra for the three Ar complexes all agree very well with the corresponding experiments, predicting the three observed bands and fairly reproducing their relative intensities. Moreover, the slightly broader feature seen for PdAu_3_^+^·Ar_5_ near 150 cm^−1^ is reproduced by a double band in the calculations, due to a symmetry breaking. The agreement between the calculations and the experiment, each with the same geometry for the metallic framework, confirms that Ar does not significantly modifies the geometry of PdAu_3_^+^. Accordingly, we conclude that the structure of the bare PdAu_3_^+^ cluster, for which the IR spectrum was not recorded, is the one in isomer 1. While Ar does not modify the isomeric configuration adopted by PdAu_3_^+^, it slightly modifies the Au–Pd bond lengths in the cluster. In PdAu_3_^+^, these are 2.660 Å, whereas in PdAu_3_^+^Ar_4_ they are 2.634 Å. IR spectra of the bare and tagged species of PdAu_3_^+^ are compared in Fig. S2 of the ESI,† with a representation of the PdAu_3_^+^Ar_4_ vibrational modes in Fig. S3.†

**Fig. 3:**
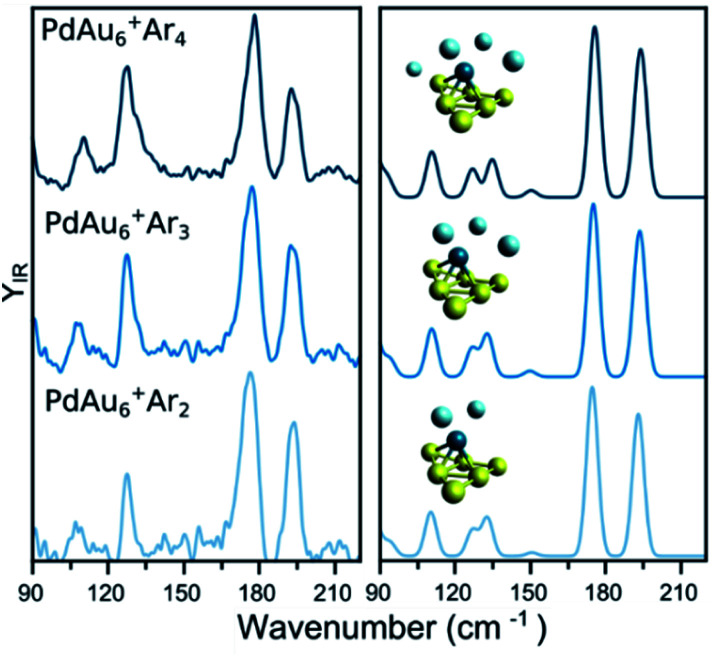
(left) Experimental IR spectra of PdAu_6_^+^Ar_*m*_ (*m* = 2, 3, 4) clusters. (right) Simulated IR spectra of the complexes, considering the lowest-energy isomer (see Fig. 5 for details).

A similar analysis on the effect of Ar attachment on the metallic framework structure is made for PdAu_6_^+^, as summarized in Fig. 3. In the left panel, the measured spectra for PdAu_6_^+^Ar_2_, PdAu_6_^+^Ar_3_, and PdAu_6_^+^Ar_4_ are depicted, with the corresponding simulated spectra using the geometry of the putative lowest-energy configuration for PdAu_6_^+^, on the right. The recorded spectra are highly similar, with bands around 110, 127, 178, and 194 cm^−1^, suggesting that Ar is not affecting the structure adopted by the metal framework. The computed spectra for isomer 1 (other isomers are discussed later), a triangular Au_6_ with Pd above the plane coordinated to four Au atoms, reproduces all observed bands. A minor difference is the band at 127 cm^−1^ for which the calculations provide a doublet feature, but the experimental data only has a shoulder. Despite this small difference, the calculations employing isomer 1 reproduce very well the experimental results, confirming also that for this cluster size the attachment of many Ar atoms does not affect the underlying geometry adopted by the metallic framework in PdAu_6_^+^.

**Fig. 4:**
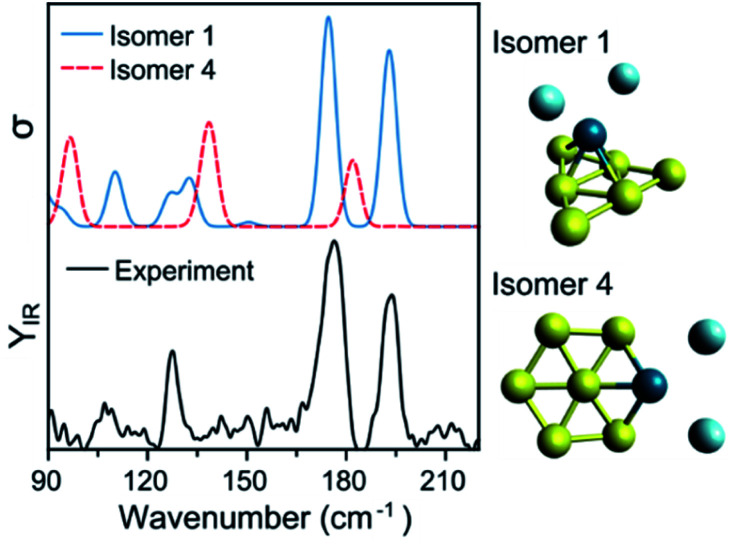
Experimental (bottom) and simulated (top) IR spectra of PdAu_6_^+^Ar_2_. Calculations for two isomers, with geometries depicted on the right of the figure.

To assign the geometry of a specific PdAu_*n*−1_^+^ cluster, the experimental data is compared with simulated IR spectra of different isomers of that size. As an example, Fig. 4 compares the experimental spectrum (bottom) with the simulated spectra (top) of two isomers of PdAu_6_^+^Ar_2_ (more isomers are discussed later). As already mentioned, the computed spectra for isomer 1 reproduce nicely the experiment. The computed spectra of isomer 4, where Pd substitutes a Au atom in Au_7_^+^,^5^ disagree with the experimental result, predicting bands at 97, 139, and 182 cm^−1^ that do not appear in the measurement. Comparing the results from both isomers, the geometry of isomer 1 is likely the one present in the molecular beam. The relative energy (Δ*E*) between the isomers also support this conclusion, as isomer 4 is found 0.63 eV higher.

Such a visual comparison between the experimental and simulated IR spectra is illustrative, and has been proven useful for identifying cluster geometries.^5,12^ If many isomers are compared, a more quantitative comparison between experiment and calculations is advisable. For this reason, we directly contrast the simulated spectra with the experimental curve, using the Kullback–Leibler (*D*_KL_) divergence, which quantifies how much two curves differ from each other. *D*_KL_ is zero for identical curves, and increases the more they diverge.^48^ Therefore, we can assign geometries by comparing *D*_KL_^−1^ values; a better agreement between experiment and calculation is reflected in a larger *D*_KL_^−1^ value. For this procedure, the computed vibrational modes of a specific isomer were used to construct a simulated IR spectrum, in which Gaussian functions were assumed centered at each vibrational frequency. These frequencies were scaled given the factor determined in ref. 5. The width of the Gaussian (5 cm^−1^) was determined by a prior visual inspection of the experimental and simulated IR spectra. The selected width was confirmed to provide the higher *D*_KL_^−1^ values. Using the benchmark analysis in Fig. 1 as an example, *D*_KL_^−1^ values of 0.08 (LC-ωPBEh), 0.05 (PBE0), and 0.04 (PBE) are calculated, indicating that LC-ωPBEh gives the best agreement. Another example of the procedure is presented in Fig. 4 for PdAu_6_^+^Ar_2_, with *D*_KL_^−1^ values of 0.14 and 0.04 obtained for isomers 1 and 4, respectively.

**Fig. 5:**
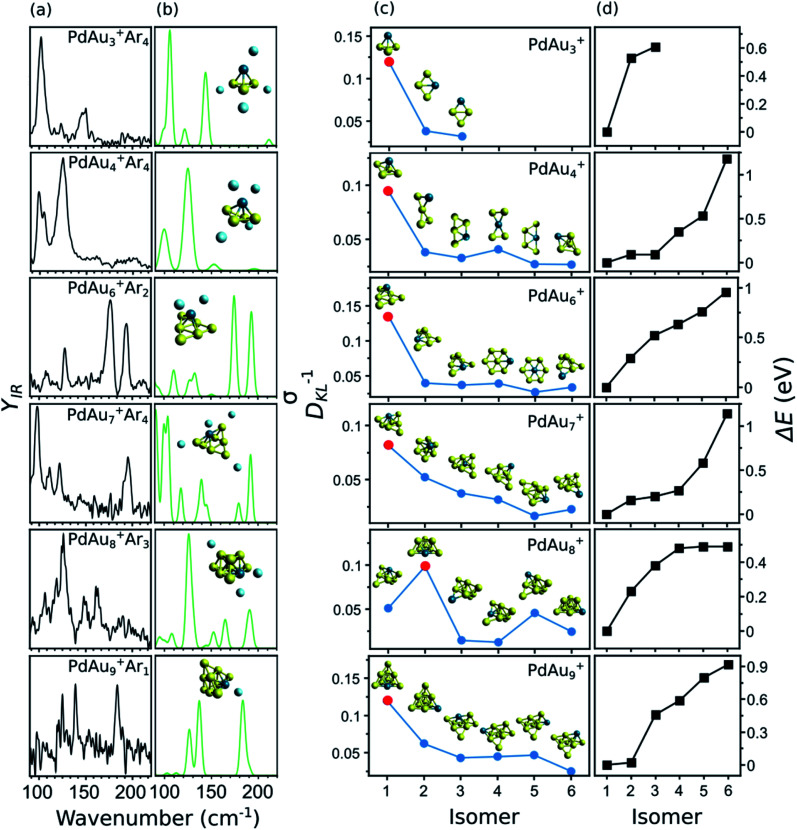
(a) Experimentally measured IR spectra of PdAu_*n*−1_^+^Ar_*m*_ clusters. (b) Calculated IR spectra of the assigned isomers. The isomers are shown as insets. (c) Calculated values of *D*_KL_^−1^, comparing the measured IR spectrum of a cluster size with those simulated for each isomer. A higher *D*_KL_^−1^ value means a better correspondence between the experimental data and the calculations. The insets show the metallic framework of each isomer, although the simulated spectra used for calculating *D*_KL_^−1^ correspond to the complexes including Ar. The *D*_KL_^−1^ value of the assigned geometry is marked by a red dot. (d) Relative energy of each isomer with respect to the putative lowest-energy configuration (iso1).

A summary of the structural assignment for the PdAu_*n*−1_^+^ (*n* = 3–5, 7–10) clusters is shown in Fig. 5, with panel (a) showing the measured IR spectra of each complex. As mentioned, the spectrum of PdAu_3_^+^Ar_4_ has three bands at 102, 123, and 147 cm^−1^. For PdAu_4_^+^Ar_4_, two intense bands are observed, centered at 125 and 99 cm^−1^, with a side peak at 105 cm^−1^. The spectrum of PdAu_6_^+^Ar_2_, already discussed, consists of four clear bands, at 110, 127, 178, and 194 cm^−1^. For PdAu_7_^+^Ar_4_, four pronounced bands are seen, with maxima at 98, 110, 122 and 195 cm^−1^, in addition to some weaker features that could be hidden under the noise level in the 130 to 180 cm^−1^ spectral range. The IR spectrum of PdAu_8_^+^Ar_3_ has three clear bands at 126, 149, and 162 cm^−1^, with possible bands at 93, 106, 118, and 187 cm^−1^. Finally, the experimental IR spectrum of PdAu_9_^+^Ar_1_, with a signal-to-noise ratio a bit lower than for the other sizes, shows three clear bands at 126, 140, and 185 cm^−1^. For comparison, Fig. 5b presents the simulated IR spectra of the identified isomers, which are assigned as follow.

In panel (c) of Fig. 5, the blue circles show the *D*_KL_^−1^ values of six isomers (except for PdAu_3_^+^) for each PdAu_*n*−1_^+^ (*n* = 4–5, 7–10) cluster. The relative energies (Δ*E*) of these isomers are given in panel (d), and are calculated with respect to isomer 1, which is the putative lowest-energy structure. As an inset in panel (c), the geometry of each considered isomer is depicted. As seen from the figure, for each cluster size, there is always one isomer that stands out for having a significantly higher *D*_KL_^−1^ value (highlighted in the figure by a red dot). This is the one we assign as the isomer present in the molecular beam. It corresponds to the putative lowest-energy structure in all cases, except for PdAu_8_^+^, for which the *D*_KL_^−1^ analysis points to isomer 2 as the geometry present in the experiment (0.23 eV above the computed lowest energy structure).

### PdAu_3_^+^

Three isomers are considered for PdAu_3_^+^Ar_4_. In isomer 1, the metal atoms adopt a 3D pyramidal geometry, whereas isomers 2 and 3 form a rhombus, with Pd adopting different coordination sites. The *D*_KL_^−1^ analysis clearly assigns isomer 1 as the species present in the molecular beam, in agreement with the high relative energies of isomers 2 and 3, being 0.53 and 0.61 eV higher than isomer 1, respectively.

### PdAu_4_^+^

In previous DFT calculations, a twisted bow tie shaped structure (isomer 2) of PdAu_4_^+^ was predicted as the lowest in energy.^31,32,34^ In the current work, however, a bipyramid geometry was found 0.16 eV lower in energy (isomer 1). The simulated IR spectrum of isomer 1 agrees much better with the experimental data than the one of isomer 2 (*D*_KL_^−1^ values of 0.10 and 0.03, respectively). The other considered isomers yield equally low *D*_KL_^−1^ values, so they can be discarded as geometries present in the molecular beam. In a previous study, the visible/near-UV absorption spectrum of PdAu_4_^+^Ar_1_ was recorded and only isomer 2 was considered in the analysis.^34^ For this isomer, the experiment did not match the time-dependent DFT calculation of the optical spectrum. The ESI includes a calculation for isomer 1 of PdAu_4_^+^·Ar_1_ that agrees better with the experiment (Fig. S4†). This further supports the current structural assignment.

### PdAu_6_^+^

As was discussed in Fig. 3, the putative lowest-energy isomer of PdAu_6_^+^ (isomer 1) is formed by a triangular Au_6_ shape with Pd above it. Other isomers are considered, as detailed in Fig. 5c, with geometries similar to isomer 1, but with Pd adopting a different coordination (isomers 2, 3, and 6), or with the same planar shape as Au_7_^+^ and Pd substituting a Au atom (isomers 4 and 5). The *D*_KL_^−1^ analysis unambiguously favors isomer 1, with a *D*_KL_^−1^ value of 0.14, while the other isomers have values close to 0.03. Isomer 1 is also the lowest in energy, with isomer 2 having a relative energy of +0.29 eV.

### PdAu_7_^+^

The structural assignment is less obvious for PdAu_7_^+^. Isomer 1, where Pd adopts a symmetrical position substituting a Au atom in Au_8_^+^,^5^ correctly predicts the mode at 195 cm^−1^, although the agreement in the low-frequency range is not perfect (by comparing panels (a) and (b) in Fig. 5). Isomer 2, instead, does well below 150 cm^−1^, but the higher frequency modes are blue shifted with respect to the experiment (see Fig. S9 in the ESI†). The *D*_KL_^−1^ analysis suggests that isomer 1 (*D*_KL_^−1^ = 0.08) is the geometry present in the experiment, however, the *D*_KL_^−1^ value of isomer 2 (*D*_KL_^−1^ = 0.05) is not that different. The values for isomers 3 to 6 are around 0.03 and those isomers can thus be disregarded. Hence, isomer 1 is assigned in view of the better correspondence with the experiment and its lower relative energy, with perhaps some contribution from isomer 2.

### PdAu_8_^+^

The lowest-energy isomer of PdAu_8_^+^Ar_3_ (isomer 1) resembles the geometry assigned to Au_9_^+^ (formed by two Au_6_ triangles sharing three Au atoms),^5^ with the substitution of the most coordinated Au atom by the Pd dopant. The agreement between the experiment and the calculation for isomer 1 is low, with a *D*_KL_^−1^ value of 0.05. Instead, the calculated spectrum of isomer 2, a two-layer cluster with a PdAu_5_ base and a Au_3_ second layer, agrees much better (*D*_KL_^−1^ = 0.10). As shown in Fig. 5b, the calculation of isomer 2 reproduces all the experimental bands, except maybe the relative intensity of the 198 cm^−1^ band and the feature at 118 cm^−1^, although the latter simply can be noise. The other isomers are not considered because of their low *D*_KL_^−1^ values.

This analysis provides strong evidence that isomer 2 is the isomer present in the molecular beam, even though it is computationally 0.23 eV less stable than isomer 1. Therefore, a study purely based on theory would incorrectly identify this cluster's geometry. We have three possible explanations for the assignment of a higher energy isomer: (1) Ar adsorption modifies the relative order of the isomeric structures. This explanation is unlikely since the calculated Ar binding energies differ by < 0.01 eV for isomer 1 and isomer 2 of PdAu_8_^+^Ar_*m*_, whose relative energy differences are 0.19, 0.19, 0.19, and 0.23 eV for *m* = 0, 1, 2, and 3, respectively. Thus, irrespective of the number of Ar atoms isomer 1 is energetically preferred over isomer 2 by about 0.2 eV. (2) The calculated relative energies are not accurate. To explore this possibility, the relative energy between the isomers was also calculated using the double-hybrid B2PLYP functional. Double-hybrid DFT functionals are typically considered more accurate for total energy calculations, at the expense of very large computing times.^49^ See for example the correct prediction of dissociation energies of Au_*n*_^+^ clusters by the B2PLYP functional.^50^ This calculation yields an energy difference of 0.20 eV, thus essentially the same value as with LC-ωPBEh. This observation, however, does not rule out the possibility that DFT itself is not appropriate in this case, although given its success for the other sizes that explanation seems unlikely. (3) During the growth process, the PdAu_8_^+^Ar_*m*_ cluster is kinetically trapped in isomer 2. Kinetic trapping is certainly possible when clusters are formed by laser ablation and gas condensation, where the ablated plasma is rapidly cooled down by collisions with the carrier gas, in combination with a rapid supersonic expansion.^51^ In consequence, the produced size distributions are in many cases different from the expected thermodynamic equilibrium.^52^ Out of the three possibilities, option 3 seems most likely, considering that the 0.23 eV energy difference between the isomers is large enough as to not be ascribed to DFT uncertainty.^53^ Moreover, as detailed in the ESI (Fig. S5†), a scan of the potential energy surface of PdAu_8_^+^, along the reaction coordinate connecting isomers 1 and 2, shows a barrier of 0.18 eV with respect to isomer 2. This barrier could well trap the cluster in the geometry of isomer 2, given the experimental conditions.

### PdAu_9_^+^

The two lower-energy isomers of PdAu_9_^+^Ar_1_ have a pyramidal structure with a different position of the Pd dopant. Four other isomers are considered, with the predicted shape of Au_10_^+^ (formed by adding a low-coordinated Au atom to Au_9_^+^) and a substitution of a Au atom by Pd, in different coordination sites. The energy difference between the first two isomers is very small (0.02 eV), but the simulated IR spectrum of isomer 1 is in much better agreement with the experiment, with a *D*_KL_^−1^ value of 0.12, in comparison with 0.06 for isomer 2. The calculation of isomer 1 predicts particularly well the bands at 126 and 140 cm^−1^, as is seen in Fig. 5b. These bands are not reproduced by the calculation of isomer 2 (Fig. S11 in the ESI†). All the other isomers agree poorly with the experiment and are found at higher relative energies. Accordingly, we assign isomer 1, which can be formed by adding a Au atom to the assigned isomer 2 of PdAu_8_^+^.

## Discussion

**Fig. 6:**
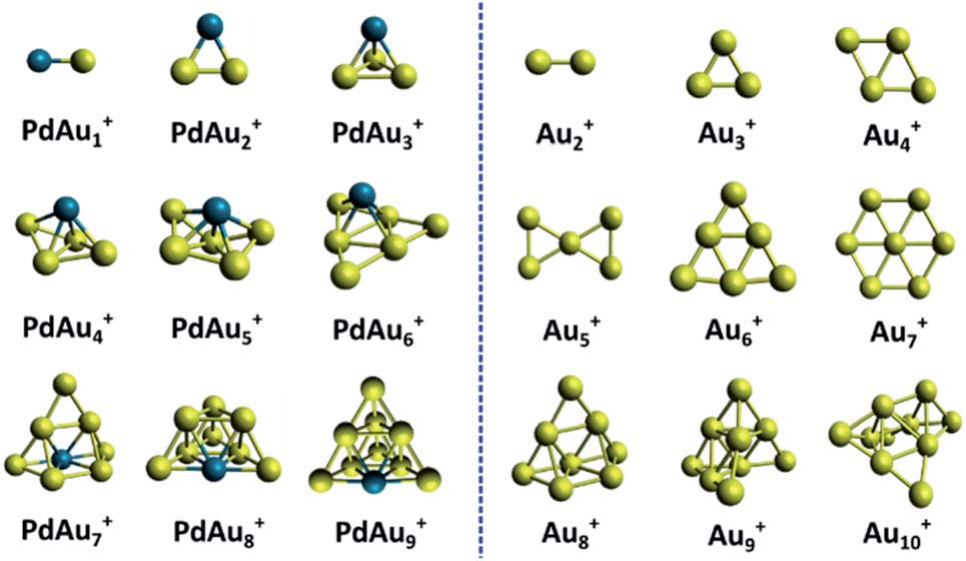
Comparison of the structures of PdAu_*n*−1_^+^ and Au_*n*_^+^ clusters. The structures of Au_*n*_^+^ and PdAu_5_^+^ are reproduced from ref. 5 and 31.

An overview of the identified PdAu_*n*−1_^+^ (*n* = 3–5 and *n* = 7–10) clusters is shown in Fig. 6. The calculated lowest energy isomers of PdAu_*n*−1_^+^ clusters with *n* = 2 (a trivial dimer) and *n* = 6 are extracted from ref. 31, and are added for completeness, even though the current study provided no experimental data for these sizes. For comparison, the figure also depicts the identified geometries for the pure Au_*n*_^+^ clusters, reproduced from ref. 5 and 31.

Comparing size-by-size, the geometries of the PdAu_*n*−1_^+^ clusters are very different from those of Au_*n*_^+^, except for *n* = 8. This is very different from other doped Au clusters, such as AgAu_*n*−1_^+^, where doping barely affects the cluster geometries.^17,39,42^ From PdAu_3_^+^, all Pd doped cationic gold clusters are unambiguously three-dimensional. This is in clear contrast with the pure Au_*n*_^+^ clusters, which are planar up to Au_7_^+^. Pd thus induces a drastic 2D to 3D transition at the smallest possible size, *n* = 4. We also note that the isomer identified for PdAu_4_^+^, which has not been considered earlier, can be formed by adding an additional Au atom to PdAu_3_^+^.

Previous studies have revealed that PdAu_6_^+^ is a particularly stable cluster, as a consequence of its closed-shell electronic configuration. To achieve such electronic configuration, the Pd dopant promotes one of its d electrons to the s shell (4d^10^ to 4d^9^5s^1^), which subsequently delocalizes over the entire cluster. Such delocalization, however, was found to strongly depend on the geometry of PdAu_6_^+^, and was only predicted for some isomers.^31^ The current structural assignment confirms the analysis of ref. 31, proving that PdAu_6_^+^ can be described as a nearly planar Au_6_ triangle, with Pd adopting a μ^4^ configuration. Substituting one Au in Au_7_^+^ by Pd drastically changes the geometry (see Fig. 6).

In ref. 31, PdAu_9_^+^ was also identified as a species with enhanced stability, with a closed-shell electronic configuration. In this case, however, only the Au atoms delocalize electrons over the cluster volume (6s^1^), in contrast with the PdAu_6_^+^ cluster. Our analysis confirms the theoretical results from ref. 31, with PdAu_9_^+^ adopting a compact pyramidal geometry with high symmetry.

The Bader charges (calculated with the Multiwfn software package;^54^ see Section 9 in ESI†) reveal that the interaction of Pd with the gold framework is significant. Despite this, the Au framework in PdAu_6_^+^ remains similar to the geometry of Au_6_^+^. A similar observation can be made for the smaller *n* < 6 clusters. For example, PdAu_3_^+^ is formed by adding the Pd atom to the Au_3_^+^ triangle in a three-fold coordinated site. PdAu_4_^+^ adopts a three-dimensional structure with Pd at the fourfold coordinated site on the rhombus Au_4_^+^, which undergoes a minor out-of-plane distortion. In PdAu_5_^+^, the Pd atom is located above the central atom of the bow tie Au_5_^+^ structure. This bow tie geometry is distorted, but still recognizable in PdAu_5_^+^. For *n* = 9 and 10, instead, the Au_*n*−1_^+^ frameworks are not recognizable in PdAu_*n*−1_^+^. This could indicate that the Au–Pd interaction is strong enough to induce an isomerization in larger Au_*n*_^+^ clusters (*n* > 7), but not for the smaller ones.

## Conclusions

The structures of small Pd doped Au clusters, PdAu_*n*−1_^+^ (*n* ≤ 10), were investigated by combining experimental infrared multiple photon dissociation spectroscopy with DFT calculations. The influence of Pd on the geometry of the pure Au_*n*_^+^ species was found to be size-dependent. PdAu_*n*−1_^+^ clusters are three-dimensional from PdAu_3_^+^, in sharp contrast with pure gold clusters, which remain planar up to Au_7_^+^. Interestingly, up to *n* = 7, the geometry of Au_*n*−1_^+^ is recognizable in PdAu_*n*−1_^+^. Instead, for *n* ≥ 9, the structures are drastically different. In PdAu_7_^+^, the Pd dopant substitutes a Au atom in Au_8_^+^, whereas for *n* = 9 and 10, the doped clusters adopt highly symmetric pyramidal shapes.

Importantly, the geometry assigned to PdAu_4_^+^, which corresponds to the putative global minima of the cluster, was not considered in previous studies that assumed geometries of PdAu_*n*−1_^+^ purely based on DFT calculations. Furthermore, the geometry determined for PdAu_8_^+^ does not correspond to the lowest-energy structure predicted by DFT, suggesting a kinetic trapping of a higher-energy isomer of this species during the formation process. In both cases, we show that theory alone cannot be used when determining the geometries of clusters, and that unambiguous assignment requires a joint experimental and computational effort.

## Conflicts of interest

There are no conflicts to declare.

## Supplementary Material

NA-003-D1NA00587A-s001
