# Supplementary material for: The size-dependent influence of palladium doping on the structures of cationic gold clusters
Source: Nanoscale Adv. 2021 Sep 21;3(21):6197–205. doi: 10.1039/d1na00587a (PMC8548875; doi:10.1039/d1na00587a)
Supplement: NA-003-D1NA00587A-s001 [file NA-003-D1NA00587A-s001.pdf]

Electronic Supplementary Material (ESI) for Nanoscale Advances.
This journal is © The Royal Society of Chemistry 2021

Supplementary Information

The size-dependent influence of palladium doping on the structures of cationic gold clusters

Piero Ferrari,[a] Laia Delgado-Callico,[b] Olga V. Lushchikova,[c] Gao-Lei Hou,[a] Francesca Baletto,[b] Joost M. Bakker[c] and Ewald Janssens[a]

[a] Quantum Solid-State Physics, Department of Physics and Astronomy, KU Leuven, Leuven, Belgium

[b] Department of Physics, King's College London, London, UK

[c] Radboud University, Institute for Molecules and Materials, FELIX Laboratory, Nijmegen, The Netherlands

Content

1. DFT functional benchmark analysis on $PdAu_2^+Ar_6$
2. Calculated infrared spectra of $PdAu_3^+Ar_m$ (m = 0-3) clusters
3. Representation of vibrational modes of $PdAu_3^+Ar_4$
4. Calculated Ar adsorption energies of $PdAu_3^+Ar_m$ (m = 1-6) clusters
5. Photodissociation and calculated optical spectra of $PdAu_4^+Ar_1$
6. Potential energy surface of $PdAu_8^+$ along the reaction coordinate connecting isomers 1 and 2
7. Measured and simulated infrared spectra
8. IR spectrum of $PdAu_5^+Ar_7$
9. Bader and Löwdin partial charges on the assigned $PdAu_n^+$ clusters
10. XYZ coordinates of selected clusters

1. DFT functional benchmark analysis on $PdAu_2^+Ar_6$

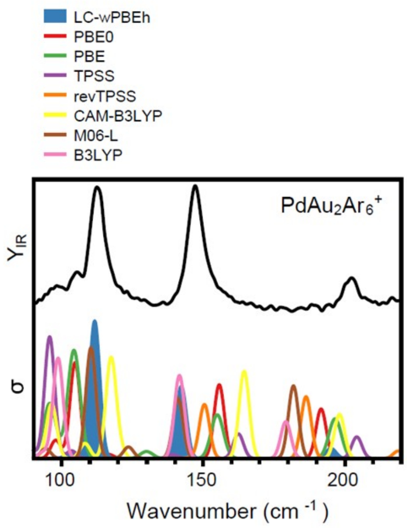

Figure S1. Experimental (top) and calculated (bottom) IR spectra of $PdAu_2^+Ar_6$. Eight functionals have been considered for the calculations (PBE, TPSS, revTPSS, M06-L, PBE0, B3LYP, CAM-B3LYP and LC-ωPBEh), with LC-ωPBEh providing the best agreement. In all cases, the Def2-TZVPP basis set was employed. Using the analysis discussed in the main article, D_{KL}^{-1} is calculated for each of the simulated spectra, giving: 0.08 (LC-ωPBEh), 0.05 (PBE0), 0.04 (PBE), 0.03 (TPSS), 0.02 (revTPSS), 0.03 (CAM-B3LYP), 0.02 (B3LYP) and 0.04 (M06-L).

2. Calculated infrared spectra of $PdAu_3^+Ar_m$ (*m* = 0-3)

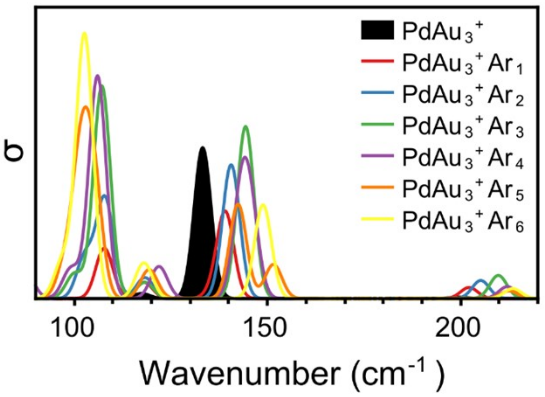

Figure S2. Calculated IR spectra of $PdAu_3^+Ar_m$ (*m* = 0-3), with the metal framework adopting the geometry of isomer 1 (see Figure 2 of main text).

3. Representation of the vibrational modes of $PdAu_3^+Ar_4$

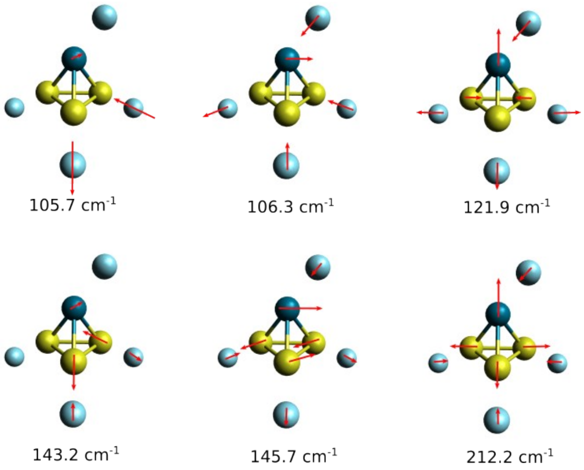

Figure S3. Visual representation of the vibrational modes of $PdAu_3^+Ar_4$ (isomer 1). The red arrows indicate the motion of the atoms.

4. Calculated Ar adsorption energies of $PdAu_3^+Ar_m$ ($m \leq 6$) clusters

Table S1. Adsorption energy of the mth attached Ar atom in $PdAu_3^+Ar_m$ ($m \leq 6$). The energy is calculated as $E_{ads} = E(PdAu_3^+Ar_m) - E(PdAu_3^+Ar_{m-1}) - E(Ar)$, with E the total energy. Harmonic zero point energy corrections are applied.

$PdAu_3^+Ar_m$	E_{ads} (eV)
0	
1	0.22
2	0.21
3	0.19
4	0.15
5	0.09
6	0.07

5. Photodissociation and calculated optical spectra of $PdAu_4^+Ar_1$

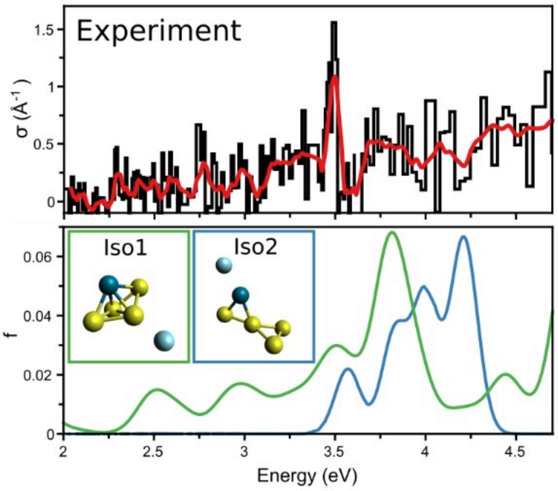

Figure S4. Top: Experimental photodissociation spectrum recorded for $PdAu_4^+Ar$. The data is reproduced from Ref. [1]. Bottom: TDDFT calculated optical spectra of the two lowest energy isomer of $PdAu_4^+Ar$: isomer 1 (green line) and isomer 2 (blue line). The calculation of isomer 2 is reproduced from Ref. [1]. The TDDFT calculation of isomer 2 was performed with the NWChem 6.8 software package [2], employing the LC-ωPBEh functional in combination with the def2-TZVPP basis set. Def2-ECP pseudopotentials were used for Au and Pd (19 and 18 valence electrons are included explicitly, respectively). All electron are accounted explicitly for Ar. This is the same method and with the same DFT software as in the work of Ref. [1].

[1] V. Kaydashev, P. Ferrari, C. Heard, E. Janssens, R. L. Johnston and P. Lievens, Part. Part. Syst. Charact. 2016, 33, 364–372.

[2] M. Valiev, E. J. Bylaska, N. Govind, K. Kowalski, T. P. Straatsma, H. J. J. Van Dam, D. Wang, J. Nieplocha, E. Apra, T. L. Windus and W. A. de Jong, Comput. Phys. Commun. 2010, 1181, 1477.

6. Potential energy surface of $PdAu_8^+$ along the reaction coordinate connecting isomers 1 and 2

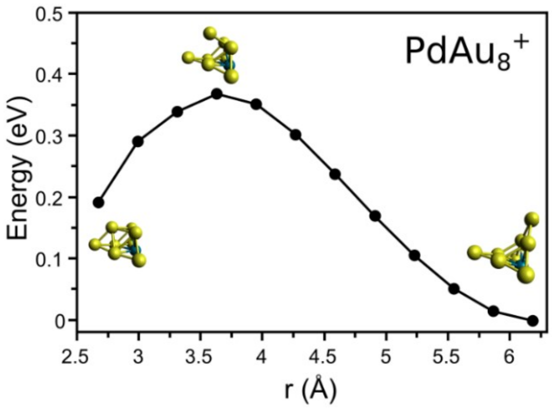

Figure S5. Scan of the potential energy surface of $PdAu_8^+$ through the reaction coordinate (r) that connects isomer 1 (right) and isomer 2 (left), with the energy of the former as the reference value (zero in energy). The calculation is performed using the same DFT method as described in the main text. In each point in the figure, the distance between the two evolving Au atoms in the reaction coordinate are varied, while all other atoms in the cluster are relaxed. From isomer 2, an energy barrier of 0.18 eV must be overcome to reach isomer 1.

7. Measured and simulated infrared spectra

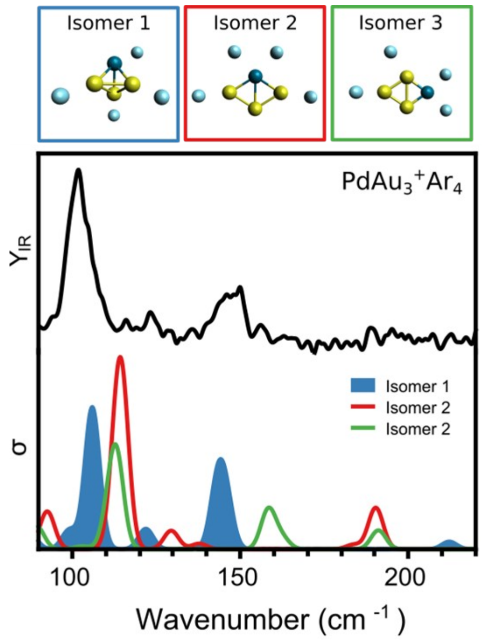

Figure S6. Experimental spectrum (top) and simulated infrared spectra of the three lowest-energy isomers (bottom) of $PdAu_3^+Ar_4$. The assigned geometry, isomer 1 (see the main text), is filled in blue. At the top of the figure, the geometries of the different isomers are depicted.

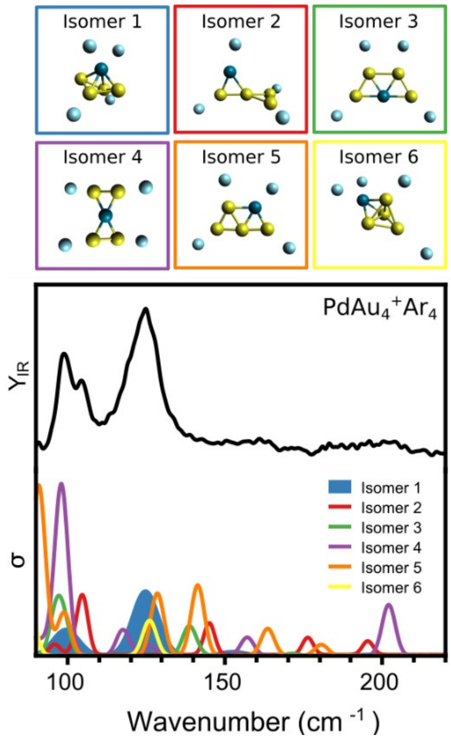

Figure S7. Experimental spectrum (top) and simulated infrared spectra of the lowest-energy isomers (bottom) of $PdAu_4^+Ar_4$. The assigned geometry, isomer 1 (see the main text), is filled in blue. At the top of the figure, the geometries of the different isomers are depicted.

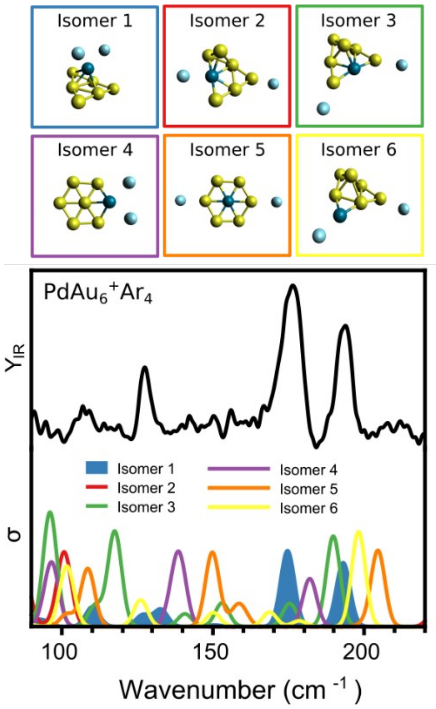

Figure S8. Experimental spectrum (top) and simulated infrared spectra of the lowest-energy isomers (bottom) of $PdAu_6^+Ar_2$. The assigned geometry, isomer 1 (see the main text), is filled in blue. At the top of the figure, the geometries of the different isomers are depicted.

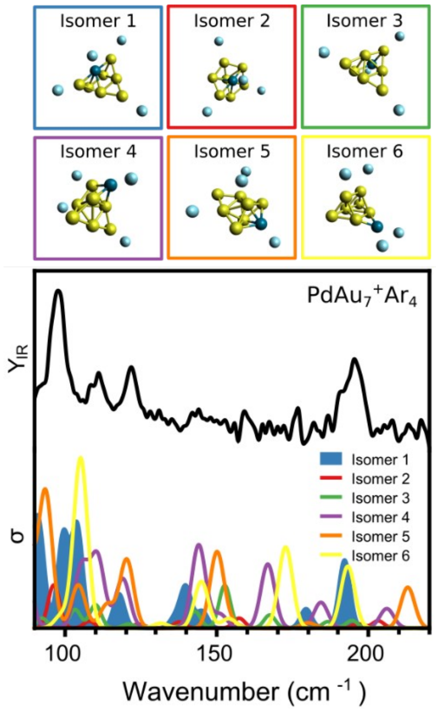

Figure S9. Experimental spectrum (top) and simulated infrared spectra of the lowest-energy isomers (bottom) of $PdAu_7^+Ar_4$. The assigned geometry, isomer 1 (see the main text), is filled in blue. At the top of the figure, the geometries of the different isomers are depicted.

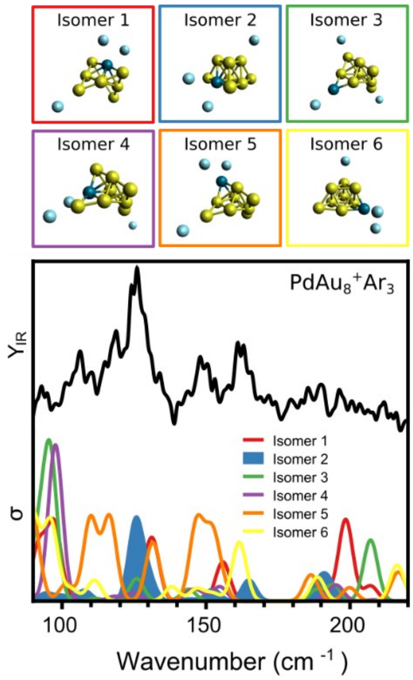

Figure S10. Experimental spectrum (top) and simulated infrared spectra of the lowest-energy isomers (bottom) of $PdAu_8^+Ar_3$. The assigned geometry, isomer 2 (see the main text), is filled in blue. At the top of the figure, the geometries of the different isomers are depicted.

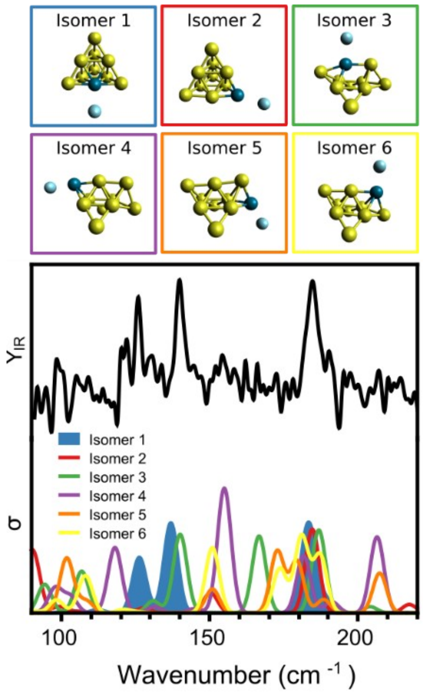

Figure S11. Experimental spectrum (top) and simulated infrared spectra of the lowest-energy isomers (bottom) of $PdAu_9^+Ar_1$. The assigned geometry, isomer 1 (see the main text), is filled in blue. At the top of the figure, the geometries of the different isomers are depicted.

8. IR spectrum of $PdAu_5^+Ar_7$

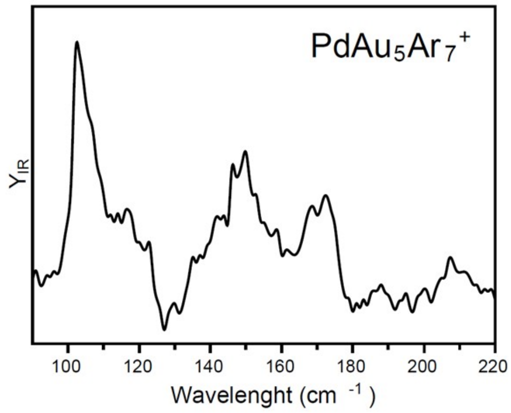

Figure S12. IRMPD spectrum of $PdAu_5Ar_7^+$.

9. Bader and Löwdin charges (in units of e) on the assigned $PdAu_n^+$ clusters

Bader charges

$PdAu_2^+$

Au	Au	Pd
0.28	0.28	0.44

$PdAu_3^+$

Au	Au	Au	Pd
0.23	0.23	0.23	0.31

$PdAu_4^+$

Au	Au	Au	Au	Pd
0.10	0.10	0.22	0.22	0.32

$PdAu_5^+$

Au	Au	Au	Au	Au	Pd
0.13	0.19	0.19	0.13	0.08	0.28

$PdAu_6^+$

Au	Au	Au	Au	Au	Au	Pd
0.07	0.12	0.12	0.12	0.10	0.10	0.37

$PdAu_7^+$

Au	Au	Au	Au	Au	Au	Au	Pd
0.14	0.07	0.11	0.09	0.09	0.11	0.14	0.25

$PdAu_8^+$

Au	Au	Au	Au	Au	Au	Au	Au	Pd
0.09	0.13	0.05	0.10	0.12	0.10	0.12	0.05	0.24

$PdAu_9^+$

Au	Au	Au	Au	Au	Au	Au	Au	Au	Pd
0.08	0.12	0.08	0.05	0.12	0.12	0.12	0.12	0.05	0.14

Löwdin charges

$PdAu_2^+$

Au	Au	Pd
0.42	0.42	0.16

$PdAu_3^+$

Au	Au	Au	Pd
0.34	0.34	0.34	-0.02

$PdAu_4^+$

Au	Au	Au	Au	Pd
0.30	0.30	0.25	0.25	-0.10

$PdAu_5^+$

Au	Au	Au	Au	Au	Pd
0.28	0.21	0.21	0.28	0.30	-0.28

$PdAu_6^+$

Au	Au	Au	Au	Au	Au	Pd
0.32	0.11	0.11	0.00	0.27	0.27	-0.08

$PdAu_7^+$

Au	Au	Au	Au	Au	Au	Au	Pd
0.22	0.26	0.02	0.32	0.32	0.02	0.26	-0.42

$PdAu_8^+$

Au	Au	Au	Au	Au	Au	Au	Au	Pd
0.29	0.08	0.32	0.16	0.32	0.03	0.16	0.03	-0.39

$PdAu_9^+$

Au	Au	Au	Au	Au	Au	Au	Au	Au	Pd
0.30	-0.01	0.30	0.33	0.05	0.05	0.05	0.05	0.33	-0.45

10. XYZ coordinates (in Å) of assigned geometries

$PdAu_2Ar_6^+$

Au	0.0305845187	-1.0315337067	-0.8728450976
Au	0.6191624260	1.3242400256	-0.0641040213
Pd	-0.9303442969	-0.2293390097	1.4177270458
Ar	-1.6453679385	1.0493584019	3.6548395986
Ar	1.2242549026	3.8095358749	0.8164402301
Ar	-0.6742019757	-3.5927376598	-1.6395700587
Ar	2.5385350261	2.4776114230	-2.4945115606
Ar	-2.4027697969	-2.3153618203	2.3424285609
Ar	1.5326881445	-1.3268445389	-3.6853584573

PdAu$_3$Ar$_4^+$

Au	-0.2993047900	1.5788218900	-0.5424055200
Au	-1.0813547700	-1.0423710800	-0.7847980100
Au	1.5751052800	-0.4263657500	-0.4590307900
Pd	-0.1587070500	-0.0870627000	1.4622614400
Ar	-0.6912054400	3.9214895900	-1.7752654600
Ar	3.9341806000	-1.0297752400	-1.5687698400
Ar	-2.6219363900	-2.5501727100	-2.3722868500
Ar	1.2830930500	1.3411817000	3.4689180700

PdAu$_4$Ar$_4^+$

Pd	-0.3856875592	1.7930832279	0.1494752023
Au	-2.1509737050	-0.1269024977	-0.6058000412
Au	2.0322169246	0.6762804184	0.6758597176
Au	-0.2940193195	-0.6316487854	1.3376726185
Au	0.4849382072	-0.2601339554	-1.3760373081
Ar	-0.5041438223	-2.5057942716	3.3044671267
Ar	0.2540195883	4.1588130461	-1.2438558615
Ar	1.3830920841	-1.5729780304	-3.5874628983
Ar	-1.8954308082	3.4612050782	1.8505509539

PdAu$_6$Ar$_2^+$

Au	0.4743676500	2.0113712000	-2.4051566500
Au	-0.6554993500	-0.2370938500	-1.6555285400
Au	1.7808738700	-0.1110275800	2.1782984700
Au	-1.6251437900	-2.5638517600	-0.9211529300
Au	1.4545674600	0.9339278200	-0.2341161800
Au	0.2832488700	-1.6161776200	0.5943018300
Pd	-0.8061429900	0.7281821800	1.1034769700
Ar	-1.5173905700	3.3140056000	1.5235577100
Ar	-2.9935912200	0.0904045100	2.5752090700

PdAu$_7$Ar$_4^+$

Au	-1.6502933600	-1.4340633700	-1.1586377900
Au	2.0295046200	1.9667722200	-1.6885326300
Au	-0.4054130200	1.0650100900	-1.3021415100
Au	-2.9125088600	0.7663744300	-0.3095367100
Au	1.3054770400	-1.1883994000	2.4545528600
Au	1.7136044100	0.0413426200	0.0660503500
Au	0.4579914800	-2.4445452400	0.2538684700
Pd	-0.8609043300	-0.2416776800	1.1395338500
Ar	-5.3955949600	2.1920313500	-0.3798306400
Ar	2.7105632100	-1.5128963800	4.9196668800
Ar	-1.2846931600	1.5380372100	3.0657536300
Ar	3.8079132900	3.8073521800	-3.1170314200

PdAu$_8$Ar$_3^+$

Au	-1.6502933600	-1.4340633700	-1.1586377900
Au	2.0295046200	1.9667722200	-1.6885326300
Au	-0.4054130200	1.0650100900	-1.3021415100
Au	-2.9125088600	0.7663744300	-0.3095367100
Au	1.3054770400	-1.1883994000	2.4545528600
Au	1.7136044100	0.0413426200	0.0660503500
Au	0.4579914800	-2.4445452400	0.2538684700
Pd	-0.8609043300	-0.2416776800	1.1395338500
Ar	-5.3955949600	2.1920313500	-0.3798306400
Ar	2.7105632100	-1.5128963800	4.9196668800
Ar	-1.2846931600	1.5380372100	3.0657536300
Ar	3.8079132900	3.8073521800	-3.1170314200

PdAu$_9$Ar$_3^+$

Au	-1.7940282700	-1.0065661500	-2.6417892100
Au	-1.9830442800	0.4069015400	-0.3799682800
Au	2.4478974300	1.8404603300	-1.0778997600
Au	0.2424940200	1.9217641400	0.4345391800

Pd	1.8445916200	-0.4021141500	0.3742635300
Au	-0.4055595700	-1.9351408000	-0.4614149100
Au	-0.5408879100	-0.3710634000	1.9626556900
Au	0.3958043600	0.3588533500	-1.9446670600
Au	0.9620862400	-2.4881433100	1.6863227400
Ar	3.4914474800	0.3261991900	2.6435040400
Ar	3.7334101000	-2.1673458600	-0.9429020700
Ar	-4.3748972813	1.2277156011	-0.4065445792

$PdAu_9Ar_1^+$

Au	-1.7940282700	-1.0065661500	-2.6417892100
Au	-1.9830442800	0.4069015400	-0.3799682800
Au	-2.0449980900	1.9265784800	1.8168171000
Au	2.4478974300	1.8404603300	-1.0778997600
Au	0.2424940200	1.9217641400	0.4345391800
Pd	1.8445916200	-0.4021141500	0.3742635300
Au	-0.4055595700	-1.9351408000	-0.4614149100
Au	-0.5408879100	-0.3710634000	1.9626556900
Au	0.3958043600	0.3588533500	-1.9446670600
Au	0.9620862400	-2.4881433100	1.6863227400
Ar	3.7334101000	-2.1673458600	-0.9429020700

Files are available at https://github.com/LaiaDelgado/AuPd
